# Psychological resilience among emergency medical teams in Singapore

**DOI:** 10.5365/wpsar.2025.16.3.1180

**Published:** 2025-07-28

**Authors:** Eunice Chan Chee Yun, Jacqueline Tan Chieh Ling, Teng Kuan Peng David, Quah Li Juan Joy, Lee Chan Yu Jimmy, Yeo Yi Wen Mathew, Pek Jen Heng

**Affiliations:** a Department of Emergency Medicine, Sengkang General Hospital, Singapore.; b Disaster Site Medical Command, Ministry of Health, Singapore.; c Department of Emergency Medicine, Tan Tock Seng Hospital, Singapore.; d Department of Emergency Medicine, Singapore General Hospital, Singapore.; e Department of Emergency Medicine, Ng Teng Fong General Hospital, Singapore.; f Department of Emergency Medicine, Khoo Teck Puat Hospital, Singapore.

## Abstract

**Problem:**

Emergency medical teams (EMTs) responding to mass casualty incidents attend to casualties in a chaotic, high-pressure and resource-limited environment that is vastly different from their day-to-day work. The nature of mass casualty incidents and the work environment can impact psychological resilience, but the psychological resilience of members of EMTs has not been evaluated.

**Context:**

In Singapore, EMTs are deployed from public hospitals, polyclinics and the Singapore Red Cross to disaster sites, where they triage, stabilize and treat casualties before evacuating them to public hospitals for further management.

**Action:**

Twenty-four members of EMTs responded to a cross-sectional survey based on a psychological resilience tool developed for health-care rescuers involved in mass casualty incidents to evaluate their psychological resilience after a full-scale exercise involving an aviation accident. Respondents completed a psychological resilience tool that was developed by experts in disaster work and research using a modified Delphi approach. There were 27 items across eight domains: optimism, altruism, preparations for disaster rescue, social support, perceived control, self-efficacy, coping strategies and positive growth.

**Outcome:**

The key observations from the survey were that (i) staff demonstrated a strong sense of altruism and had good social support; (ii) staff were not confident about their preparedness, and this led to a lack of optimism, perceived control and ability to deal with emotions; and (iii) it was necessary for respondents to reflect on their experience to find meaning to support growth after the deployment.

**Discussion:**

Optimizing casualty survival and outcomes during mass casualty incidents requires not only excellent procedural training and robust standard operating procedures and work processes but also dedicated efforts to enhance the psychological resilience of members of EMTs.

## PROBLEM

Health-care professionals are known to have higher risks of mental illness, substance abuse and suicide compared with the general population. (*1*) This is attributed to their continual exposure to high-pressure environments and elevated levels of stress at work. Psychological resilience, defined as the ability to mentally and emotionally adapt to adversity, is crucial in maintaining personal and professional well-being, thereby protecting against occupational burnout and post-traumatic stress disorder. (*2*, *3*)

There is increasing awareness that health-care professionals experience a variety of psychological consequences when responding to mass casualty incidents (MCIs), in which the number of casualties can overwhelm health-care resources and make it challenging to treat every person. (*4*) For instance, health-care professionals responding to outbreaks of Ebola virus disease experienced significant psychological distress. (*5*) Similarly, health-care professionals responding to disasters face considerable mental health challenges, including burnout and post-traumatic stress disorder, which can persist long after the event. (*6*) The literature highlights the necessity for psychological resilience in members of emergency medical teams (EMTs) to help them maintain their performance under extreme conditions.

EMTs must fulfil their roles and responsibilities in unfamiliar environments or potentially hazardous settings that are significantly different from their usual workplace. They also need to deliver care while following standard operating procedures related to medical operations and communications, which may be unfamiliar to them. As such, the nature of MCIs coupled with unfamiliarity with the work environment and work processes can negatively influence the well-being of members of EMTs and impact their response at a disaster site.

While numerous studies have looked at psychological resilience among health-care workers in the hospital setting, notably during outbreaks of diseases such as Ebola virus disease and COVID-19, very few studies have looked specifically at psychological resilience in members of EMTs responding to MCIs. (*4*-*7*) Hence, this study aimed to evaluate psychological resilience among members of EMTs deployed to MCIs in Singapore.

## CONTEXT

During MCIs, the Singapore Civil Defence Force, an emergency medical service, activates EMTs from public hospitals, polyclinics and the Singapore Red Cross and directs them to a designated area at the disaster site, where casualties are triaged, stabilized and treated before being evacuated to public hospitals for further management.

To prepare for disaster response, EMTs undergo a 1-day Disaster Medical Responder’s Course, which covers disaster response operations and processes; roles and responsibilities; command, control and communications; as well as supplies and resources. The goal is to enhance EMTs’ preparedness and operational readiness so that they can perform effectively at a disaster site and optimize outcomes for casualties.

Although psychological resilience is recognized as an important element of disaster preparedness and operational readiness, the curriculum of the Disaster Medical Responder’s Course includes only one learning outcome related to it, and this focuses on describing psychological support for members of EMTs. This content is delivered through a single didactic lecture covering self-preparation, recognizing emotions and stress reactions, and providing support for oneself and colleagues.

The limited emphasis on psychological resilience in EMT training may leave members of the teams inadequately prepared to manage the intense psychological demands of a disaster response. Without structured training to strengthen their psychological resilience, members of EMTs may struggle with decision-making and emotional regulation, and sustain long-term impacts on their psychological well-being. These could potentially affect the overall effectiveness of their response efforts during MCIs.

## ACTION

To determine whether improvements or modifications to psychological resilience training during the Disaster Medical Responder’s Course are necessary, we conducted a cross-sectional survey to evaluate the pschological resilience of members of EMTs. The findings will help identify gaps in training so that these may be addressed by incorporating specific content into the curriculum, such as training modules, practical exercises or workshops related to psychological resilience.

The survey was conducted on 30 January 2024, immediately after a full-scale exercise. The scenario involved an aircraft mishap during a flying display, resulting in a crash among spectators. The casualties were simulated with the use of standardized patients and high-fidelity mannequins with moulage. Four EMTs – three from public hospitals and one from the Singapore Red Cross – were deployed and worked closely with the Incident Manager and paramedics from the Singapore Civil Defence Force to manage casualties at the disaster site.

The survey was based on a psychological resilience tool for health-care rescuers involved in MCIs that was developed by Mao et al. using a modified Delphi approach, which included experts in disaster work and research. (*8*) This tool was the first devised to measure psychological resilience during MCIs. The tool consists of 27 items measured across eight domains: optimism (O), altruism (A), preparations for disaster rescue (P), social support (SS), perceived control (PC), self-efficacy (SE), coping strategies (CS) and positive growth (PG). Respondents rated each item on a 5-point Likert scale. Information about demographics, work experience and training for MCIs, as well as experience with exercises or actual MCIs was also collected.

## OUTCOME

The survey targeted all 32 members of the four EMTs, and 24 (75%) completed it. Among the respondents, the median age was 33 years (interquartile range [IQR]: 30–37), with 12 (50.0%) females, 7 (29.2%) males and 5 (20.8%) who chose not to disclose their gender. They had been working for a median of 10 years (IQR: 7–12). Fourteen (58.3%) had attended the Disaster Medical Responder’s Course; 12 (50.0%) had taken part in exercises previously; and 2 (8.3%) had responded to actual MCIs. The results of the survey are shown in (**Table 1**).

**Table 1:** Results of a survey of psychological resilience among emergency medical teams after a disaster exercise (*n* = 24), Singapore, 30 January 2024

Domain	Statement	Mean ± standard deviation^a^
**Optimism (O)**	**O1: I think that difficulties are everywhere during and after rescue work.**	**4.04 ± 0.75**
	**O2: I tend to think that problems confronted before, during and after deployment will be solved.**	**3.92 ± 0.78**
	**O_3_: I know that I will bounce back and get better no matter how difficult the situation is, with help from others.**	**4.33 ± 0.56**
**Altruism (A)**	**A1: I have a desire to help the victims/survivors after a disaster has occurred.**	**4.38 ± 0.58**
	**A2: I am honoured to work on the front line to offer my help to those who are affected by disaster.**	**4.54 ± 0.51**
	**A3: I feel that it is a personal responsibility to help others after disasters.**	**4.50 ± 0.59**
**Preparations for disaster rescue (P)**	**P1: I am certain of my safety and that of my family while I am deployed.**	**3.92 ± 0.88**
	**P2: I have sufficient knowledge to assess disaster risks and have disaster rescue skills, such as medical rescue skills, knowledge of psychological first aid, ethical rules and field survival skills.**	**3.79 ± 0.78**
	**P3: I am emotionally well prepared for disaster rescue.**	**3.79 ± 0.88**
	**P4: I feel physically unprepared for disaster relief.**	**2.54 ± 1.14**
**Social support (SS)**	**SS1: My family will provide me with strong support during and after my disaster relief work.**	**4.21 ± 0.78**
	**SS2: Coworkers will help me to overcome challenges at the disaster site.**	**4.54 ± 0.51**
	**SS3: I have some close friends who will provide me with much encouragement.**	**4.42 ± 0.65**
	**SS4: My work unit will provide support to my family and to me, if necessary, when I work in disaster sites.**	**4.21 ± 0.66**
**Perceived control (PC)**	**PC1: How things go during and after deployment will depend on my own actions.**	**3.88 ± 0.85**
	**PC2: I can handle various situations at a disaster site.**	**3.79 ± 0.72**
	**PC3: I can remain calm during a disaster rescue.**	**4.04 ± 0.75**
**Self-efficacy (SE)**	**SE1: I feel confident that my clinical skills are (will be) of good use for disaster work.**	**4.29 ± 0.55**
	**SE2: I can cope well with unexpected problems during disaster rescues.**	**3.92 ± 0.72**
	**SE3: I am a competent rescue worker.**	**4.13 ± 0.68**
**Coping strategies (CS)**	**CS1: I always try to find ways to address problems during disaster events.**	**4.13 ± 0.54**
	**CS2: When a victim’s/survivor’s life is threatened, I lose my temper and blame others.**	**2.00 ± 1.02**
	**CS3: I am willing to express my emotions to others if I am upset.**	**3.50 ± 0.83**
**Positive growth (PG)**	**PG1: I have gained insight about life from rescue work.**	**4.29 ± 0.55**
	**PG2: I tend to see rescue work as a challenge after deployment.**	**3.63 ± 1.06**
	**PG3: I find meaning in my deployment.**	**4.50 ± 0.51**
	**PG4: After returning from deployment to a disaster site, I have a more harmonious family life.**	**3.79 ± 0.78**

^a^ Respondents rated each item on a 5-point Likert scale.

Key observations from the responses, including mean values and standard deviations, are summarized below.

Staff demonstrated a strong sense of altruism, based on their responses to statements about having a desire to help (A1: 4.38 ± 0.58), feeling honoured to work on the front lines (A2: 4.54 ± 0.51) and believing it is their personal responsibility to help others (A3: 4.50 ± 0.59). Additionally, they had good social support for their involvement in MCIs from family  (SS1: 4.21 ± 0.78), coworkers (SS2: 4.54 ± 0.51), friends (SS3: 4.42 ± 0.65) and work units (SS4: 4.21 ± 0.66).
Staff were not confident about their preparedness in terms of physical (P4: 2.54 ± 1.14) and emotional well-being (P3: 3.79 ± 0.88); safety (P1: 3.92 ± 0.88); MCI-specific knowledge, skills and attitudes (P2: 3.79 ± 0.78; SE1: 4.29 ± 0.55; SE3: 4.13 ± 0.68); as well as coping strategies (SE2: 3.92 ± 0.72; CS1: 4.13 ± 0.54). This lack of confidence negatively impacted their optimism when encountering difficulties (O1: 4.04 ± 0.75), solving problems (O2: 3.92 ± 0.78) and bouncing back (O_3_: 4.33 ± 0.56). They also perceived a lack of control in handling problems (PC1: 3.88 ± 0.85) and solving problems (PC2: 3.79 ± 0.72) while remaining calm (PC3: 4.04 ± 0.75), as well as a lack of ability to manage and express their emotions (CS2: 2.00 ± 1.02; CS3: 3.50 ± 0.83).
The survey indicated that after deployment, efforts must be extended to help staff reflect on their experience to gain insights (PG1: 4.29 ± 0.55), overcome challenges (PG2: 3.63 ± 1.06) and find meaningful personal growth (PG3: 4.50 ± 0.51) and interpersonal relations (PG4: 3.79 ± 0.78).

## Discussion

Psychological resilience sustains clinical decision-making, improves team dynamics, and promotes personal and professional development for members of EMTs during MCIs. (*9*) Using the observations from this survey, we propose elements of a training framework for psychological resilience.

This training framework for increasing psychological resilience should be anchored in altruism and social support, which are strong, innate, protective factors for psychological resilience: staff should remind themselves of their beliefs and those who share them. (*10*, *11*) The goal is to empower EMT staff to use their MCI experience for personal and professional growth: staff should look beyond the deployment and identify takeaways for their work and life. (*11*) Training sessions should focus on self-preparedness to enhance physical and emotional well-being and safety, as well as equipping team members with MCI-specific knowledge and skills. This could be achieved using simulations to replicate the realism of MCIs, while challenging staff to apply their knowledge and skills in a physically demanding, high-stress and time-sensitive situation. In this framework, team members would be given time and space to identify issues, communicate with each other and solve problems. A team-based approach would underscore the importance of social networks within EMTs and the value of peer support. (*12*) This would improve the competence and confidence of not just the individual but also the entire EMT as members work collaboratively. (*13*) Coping strategies – such as mindfulness practices, learning to handle stress, managing conflict and psychological first aid – could also be incorporated into simulation sessions so staff can understand how psychological resilience can enhance their ability to deal with the unique challenges of MCIs. (*14*, *15*)

The limitations of this work include the fact that it is a single-country study with a small sample size, which affects the applicability of our suggested training framework for psychological resilience to other settings. The survey was also done following the EMTs’ participation in a full-scale exercise instead of an actual MCI, so the results are based on perceptions formed through simulation training rather than real-world experience. Furthermore, we did not analyse the responses for correlations between scores and characteristics of the members of the EMTs, such as demographics or experience. For instance, the presumed positive effect of participation in an actual MCI on the various domains of this psychological resilience tool could not be ascertained because only two participants had been involved in an actual MCI. Last, the training framework has yet to be developed into a detailed programme, and therefore its impact on psychological resilience is to be determined. Future research can incorporate qualitative methods, such as focus group interviews, to gain a more in-depth understanding of psychological resilience and training needs.

In conclusion, psychological resilience can positively impact the competence, confidence, experience and camaraderie of members of EMTs during MCIs. Through this work, we evaluated the psychological resilience of members of Singaporean EMTs deployed to MCIs and identified current gaps. A training framework should be proposed to incorporate psychological resilience training during the Disaster Medical Responder’s Course.
